# Biological Activities of the Essential Oil from *Erigeron floribundus*

**DOI:** 10.3390/molecules21081065

**Published:** 2016-08-13

**Authors:** Riccardo Petrelli, Giuseppe Orsomando, Leonardo Sorci, Filippo Maggi, Farahnaz Ranjbarian, Prosper C. Biapa Nya, Dezemona Petrelli, Luca A. Vitali, Giulio Lupidi, Luana Quassinti, Massimo Bramucci, Anders Hofer, Loredana Cappellacci

**Affiliations:** 1 School of Pharmacy, University of Camerino, Camerino 62032, Italy; luca.vitali@unicam.it (L.A.V.); giulio.lupidi@unicam.it (G.L.); luana.quassinti@unicam.it (L.Q.); massimo.bramucci@unicam.it (M.B.); loredana.cappellacci@unicam.it (L.C.); 2 Department of Clinical Sciences, Section of Biochemistry, Polytechnic University of Marche, Ancona 60131, Italy; orsomando@univpm.it (G.O.); l.sorci@univpm.it (L.S.); 3 Department of Medical Biochemistry and Biophysics, Umeå University, Umeå 90187, Sweden; farahnaz.ranjbarian@umu.se (F.R.); anders.hofer@umu.se (A.H.); 4 Laboratory of Medicinal Plant Biochemistry, Food Science and Nutrition, Department of Biochemistry, Faculty of Sciences, University of Dschang, PO Box 67, Dschang, Cameroon; prbiapa@yahoo.fr; 5 School of Biosciences and Veterinary Medicine, University of Camerino, Camerino 62032, Italy; dezemona.petrelli@unicam.it

**Keywords:** *Erigeron floribundus*, essential oil, antimicrobial, NadD, *Trypanosoma brucei*, cytotoxicity, antioxidant, limonene, caryophyllene oxide

## Abstract

*Erigeron floribundus* (Asteraceae) is an herbaceous plant widely used in Cameroonian traditional medicine to treat various diseases of microbial and non-microbial origin. In the present study, we evaluated the in vitro biological activities displayed by the essential oil obtained from the aerial parts of *E. floribundus*, namely the antioxidant, antimicrobial and antiproliferative activities. Moreover, we investigated the inhibitory effects of *E. floribundus* essential oil on nicotinate mononucleotide adenylyltransferase (NadD), a promising new target for developing novel antibiotics, and *Trypanosoma brucei*, the protozoan parasite responsible for Human African trypanosomiasis. The essential oil composition was dominated by spathulenol (12.2%), caryophyllene oxide (12.4%) and limonene (8.8%). The *E. floribundus* oil showed a good activity against *Staphylococcus aureus* (inhibition zone diameter, IZD of 14 mm, minimum inhibitory concentration, MIC of 512 µg/mL). Interestingly, it inhibited the NadD enzyme from *S. aureus* (IC_50_ of 98 µg/mL), with no effects on mammalian orthologue enzymes. In addition, *T. brucei* proliferation was inhibited with IC_50_ values of 33.5 µg/mL with the essential oil and 5.6 µg/mL with the active component limonene. The essential oil exhibited strong cytotoxicity on HCT 116 colon carcinoma cells with an IC_50_ value of 14.89 µg/mL, and remarkable ferric reducing antioxidant power (tocopherol-equivalent antioxidant capacity, TEAC = 411.9 μmol·TE/g).

## 1. Introduction

*Erigeron floribundus* (Kunth) Sch. Bip. (Asteraceae) is an herbaceous plant, 1.5 m in height, with pubescent, lanceolate leaves and flowers in yellowish panicles. In Cameroon, it is commonly found as a weed along roadsides, and it is widely used in folk medicine to treat angina, female infertility, AIDS, dental pain, headache and various diseases of microbial and non-microbial origin [1,2,3,4,5,6]. The leaf aqueous extract was proven to possess analgesic, anti-inflammatory and immunomodulatory activity [1,2], the dichloromethane extract inhibition against dermatophytes [7], and the leaf ethanolic and pentane extract antiplasmodial activity [8]. Despite the interest in its bioactivity, the plant has received little phytochemical investigation. With regard to the essential oil, a previous work performed on Cameroonian plants showed sesquiterpene hydrocarbons as the major essential oil constituents [9].

Given the wide use of the plant in traditional medicine, we evaluated the in vitro biological activities displayed by the essential oil obtained from the aerial parts of *E. floribundus*, namely the antioxidant, antiproliferative and antimicrobial activities that were investigated by 2,2-diphenyl-1-picrylhydrazyl (DPPH), 2,2'-azino-bis(3-ethylbenzothiazoline-6-sulphonic acid (ABTS), ferric reducing antioxidant power (FRAP), 3-(4,5-dimethyl-2-thiazolyl)-2,5-diphenyl-2H-tetrazoliumbromide (MTT), and agar disc diffusion and microdilution methods, respectively.

Because essential oils represent an interesting alternative approach against the occurrence of drug resistance in many infectious bacterial pathogens [10] and fighting protozoan parasites [11], we completed the work by investigating the activity of *E. floribundus* essential oil on nicotinate mononucleotide adenylyltransferase (NadD) and *Trypanosoma* *brucei*. Being natural in origin, essential oils and their components are believed to be environmentally favourable and user-friendly [12]. Moreover, using innovative technologies, such as nanoencapsulation, many limits of their application in medicine (mainly low water solubility and stability, high volatility and side effects) could be overcome [13].

NadD occupies a central position in bacterial NAD^+^ biosynthesis and is required for both de novo and salvage routes to generate NAD^+^ [14,15,16]. NadD has been recognized as a promising new target for developing novel antibiotics due to its crucial role in synthesizing NAD^+^ [17,18,19,20]. Another attractive aspect of targeting NadD is that it is highly conserved in the overwhelming majority of bacterial species including most pathogens. Therefore, drugs developed based on the inhibition of NadD have the potential of possessing wide-spectrum antibacterial activity.

*T. brucei* is a protozoan parasite responsible for Human African trypanosomiasis (HAT), a neglected disease also known as African sleeping sickness. HAT threatens primarily rural populations and is fatal unless treated. To date, there are a few drugs approved, although none of them are satisfactory, due to treatment failures and toxicity, and the parenteral administration that is inappropriate in settings with poor medical infrastructure. Therefore, there is an urgent need to improve HAT treatment by enhancing the oral administration and the discovery and development of cost-effective new drugs. Drug discovery efforts are nowadays directed towards natural products and medicinal plants represent a validated source for discovery of new lead compounds and standardized herbal medicines against trypanosomiasis [21].

## 2. Results

### 2.1. Essential Oil Analysis

The composition of the essential oil hydrodistilled from the aerial parts of *E. floribundus* is reported in Table 1. GC-MS analysis revealed that the essential oil had a complex chemical profile. Separation of volatile components was accomplished by using both polar and apolar columns (HP-5 MS and DB-WAX, respectively). A total of eighty-five constituents were identified accounting for 78.6% of the total composition. The main fraction of the essential oil was given by sesquiterpenes (60.4%), which were represented by oxygenated compounds (38.5%) and hydrocarbons (21.9%). Main representatives of these groups were caryophyllene oxide (12.4%) and spathulenol (12.2%), and (*E*)-β-farnesene (5.5%) and (*E*)-caryophyllene (4.2%), respectively. Monoterpenes constituted a minor part of the oil (13.0%), with hydrocarbons (11.5%) as the predominant components. Among them, limonene was the most representative (8.8%).

### 2.2. Cytotoxicity Assessment

General cell toxic effects of *E. floribundus* essential oil were evaluated using the MTT assay against the following human tumour cell lines: A375 human malignant melanoma cell line, MDA–MB 231 human breast adenocarcinoma cell line, and the HCT116 human colon carcinoma cell line. All cell lines were submitted to increasing concentrations of essential oil for 72 h. The results, collected in Table 2, show that essential oil exhibited a significant growth inhibition against the examined human cancer cells and induced a concentration-dependent inhibitory effect in the dilutions ranging from 0.78–200 μg/mL. The IC_50_ values of the essential oil were 14.9, 20.8, and 22.5 μg/mL on HCT116, MDA–MB 231, and A375 cell lines, respectively.

### 2.3. Antimicrobial Activity

The essential oil of *E. floribundus* showed a good antimicrobial activity against *Staphylococcus aureus* using both disc diffusion and microdilution methods. The inhibition zone diameter (IZD) of almost 15 mm was indicative of a good activity. The corresponding minimum inhibitory concentration (MIC) was equal to 512 µg/mL (Table 3). *S. aureus* is Gram-positive as well as *Enterococcus faecalis*. The latter was also included in the panel of tested bacteria but resulted in being less susceptible to the essential oil than *S. aureus* (IZD ≅ 9 mm) as confirmed by the much higher MIC value (4096 µg/mL). None of the Gram-negative bacterial species, namely *E. coli* and *P. aeruginosa*, were inhibited as assessed by the disc diffusion test. Therefore, antimicrobial susceptibility by the microdilution method was not investigated. In contrast, the yeast *C. albicans* growth was inhibited by the essential oil (IZD = 9 mm), which showed a minimum inhibitory concentration of 512 µg/mL by the microdilution method.

In view of the appreciable activity against *S. aureus*, an additional series of *S. aureus* clinical isolates from catheter-associated infections was selected to confirm the observation and to prove the extendibility of the antimicrobial activity of the essential oil beyond the reference strain ATCC 29213. Four isolates were selected based on their proven resistance towards oxacillin, the congener of methicillin [25]. Hence, the four isolates were MRSA (methicillin-resistant *Staphylococcus aureus*). The MICs of essential oil against the clinical isolates were substantially superimposable to the one obtained against the reference strain, differing on average by one dilution (MIC ≅ 1024 µg/mL).

### 2.4. NadD Inhibition Analysis

In our effort to explore natural compounds against nicotinate mononucleotide adenylyltransferase of the NadD family [26,27], an essential enzyme of NAD biosynthesis in most bacteria, we evaluated the in vitro inhibition of *E. floribundus* essential oil against NadD from *S. aureus*. A preliminary single point inhibition test at the concentration of 80 μg/mL indicated a ~60% inhibition with no effect on the ubiquitous mammalian isoform NMNAT1 [28]. Motivated by these results and by an observed broad-spectrum antimicrobial activity based on the diffusion IZD and MIC methods (Table 3), we performed a dose-response inhibition using an oil concentration range of 5–160 μg/mL. The IC_50_ value of the essential oil for *Sa*NadD was 98 ± 11 μg/mL.

### 2.5. Antioxidant Activity

Despite the interest on the bioactivity of *E. floribundus* based on its uses in Cameroonian traditional medicine, we evaluated for the first time the antioxidant properties of the essential oil. Antioxidant activity is a complex process usually occurring through several mechanisms and its evaluation is often carried out by more than one test method [29]. In this study, three antioxidant assays, namely DPPH free radical scavenging activity, ABTS radical cation scavenging activity, and ferric reducing antioxidant power (FRAP) were applied to evaluate the antioxidant properties of *E. floribundus* essential oil. As reported in Table 4, the antioxidant activity determined by DPPH radical scavenging ability showed a value of 16.9 ± 0.1 μmol·(Trolox equivalents, TE)/g, with an IC_50_ value about 250 times higher than that of the positive control Trolox. Higher antioxidant activity was detected in the ABTS radical cation scavenging activity assay, with an average TE value of 85.5 ± 3.6 μmol·TE/g, and an IC_50_ value about 46 times higher than that of Trolox. It is noteworthy that the essential oil appeared to act as a good reducing agent (FRAP activity, TEAC = 411.9 ± 2.40 μmol·TE/g).

### 2.6. Inhibition of T. brucei Proliferation

A test of the *E. floribundus* oil against *T. brucei* proliferation showed that it was active at a much lower concentration than the Balb/3T3 mouse fibroblasts used as a control (Table 5). When analyzing some of the components in the oil, we found that limonene had a six times higher activity against *T. brucei* than the oil itself (IC_50_ = 5.6 µg/mL) indicating that this is the major antitrypanosomal agent in *E. floribundus* oil. A calculation based on that limonene represents 8.8% of the oil and gives six times higher inhibition than the oil itself, which indicates that about half of the antitrypanosomal activity in the extract comes from limonene.

## 3. Discussion

Only a few papers dealing with the chemical composition of *E. floribundus* essential oil are available in literature. From a comparison of our data with those of Kuiate et al. [9], we notice significant differences mainly from a quantitative point of view. In that study, the sesquiterpene hydrocarbons (39.9%–74.8%) were revealed as predominant over oxygenated compounds (7.0%–13.8%). The major representatives of these groups were the same but they occurred in different amounts, i.e., (*E*)-caryophyllene (8.5%–20.1%), (*E*)-β-farnesene (14.6%–24.1%), caryophyllene oxide (0.8%–5.4%) and spathulenol (1.0%–4.1%). Similarly, therein the monoterpenes hydrocarbons constituted a small fraction (5.2%–17.3%), with limonene as the most abundant compound (2.5%–11.4%). In addition, the presence of polyacetylenic constituents such as (*E*)-2-lachnophyllum ester (3.4%–26.8%) was reported. We assume that the above differences may depend on chemical polymorphism of the species and different periods of collection.

This work reports on the cytotoxic activity of *E. floribundus* essential oil. A compound or few compounds do not emerge from the composition of essential oil, which can be responsible for the cytotoxic activity on human tumor cell lines. Caryophyllene oxide provided evidence of potent cytotoxic activity against HepG2, AGS, HeLa, SNU-1, and SNU-16 cells, with IC_50_ values of 3.95, 12.6, 13.55, 16.79, and 27.39 μM, respectively [30]. Spathulenol was reported weakly active on human epidermoid carcinoma (KB) and inactive on human breast cancer (BC) and human small cell lung cancer (NCIH187) cell lines [31]. In addition, (*E*)-caryophyllene showed weak activity on the cell lines tested, with IC_50_ values ranging from 43.27 to 67.14 µg/mL for MDA–MB 231 and A375, respectively [32]. Our previous study has demonstrated that limonene showed antiproliferative activity on the same cell lines as tested above with IC_50_ values ranging from 18.4 to 124.0 µg/mL, respectively [33]. Limonene also induces apoptosis in LS174T colon cancer cells and in a lymphoma cell line (35 µg/mL, IC_50_) [34,35]. Moreover, it shows antitumor activity on lung adenocarcinoma A549 (0.098 µL/mL, IC_50_) and hepatocarcinoma HepG2 cells (0.150 µL/mL, IC_50_) [36]. To our knowledge, data reporting the cytotoxic activity of (*E*)-β-farnesene are missing. However, the concentrations of caryophyllene oxide (12.4%), spathulenol (12.2%), limonene (8.8%), (*E*)-β-farnesene (5.5%) and (*E*)-caryophyllene (4.2%) cannot fully explain the cytotoxic activity of *E. floribundus* essential oil, which means that some other minor compounds contributed to the activity of the essential oil

The antimicrobial activity investigation revealed that the essential oil is mainly active against the Gram-positive bacteria. A special emphasis might be put on the results obtained with *S. aureus*. Both the reference strain and the methicillin-resistant clinical isolates from invasive infections were susceptible to the essential oil. Given the importance of *S. aureus* in human medical microbiology and food microbiology, the result is an important outcome. To our knowledge, this is the first study reporting on the antibacterial activity of the essential oil from *E. floribundus*. On the contrary, one single previous study investigated its antifungal activity [9]. Kuiate and coll. obtained a MIC equal to about 2 mg/mL against *C. albicans*, while in the present investigation the essential oil was inhibiting the yeast growth at 0.512 mg/mL. Considering the differences in composition outlined above, it can be possible to speculate on the putative constituents contributing to the anti-*Candida* activity. Candidates might be spathulenol and caryophyllene oxide. The hypothesis on the first is not sustained by the literature where spathulenol is described solely as possessing anti-inflammatory properties. Caryophyllene oxide was instead already investigated for its antifungal properties and is approved by the FDA as a food and cosmetic preservative [37].

On the bacterial side, a hypothesis may be done with hexadecanoic acid: its concentration in the oil (2.5%) could be compatible with its reported anti-staphylococcal activity [38].

Of note, the in vitro inhibition of NadD focusing on isolating individual components of the above oil mixtures that retain both such activities. 

From radical scavenging activity assays (i.e., DPPH and ABTS, Table 4), we found that the *E. floribundus* essential oil displayed a weak activity. The chemical profile of the oil, with spathulenol (12.2%), caryophyllene oxide (12.4%) and limonene (8.8%) as the major components, may partially justify the low inhibition on these radicals [39,40,41]. On the other hand, the high value of reducing power indicated that the essential oil components are able to act as electron donors and reduce the oxidized intermediate of lipid peroxidation so that they can act as primary and secondary antioxidants. Further studies are required to confirm the potential of this oil as reducing agent.

To the best of our knowledge, this is the first report on trypanocidal activity of the essential oil of *E. floribundus*, which further confirms the medical value of the plant. With an IC_50_ value of 5.6 µg/mL the lipophilic hydrocarbon limonene showed a quite remarkable trypanocidal activity against *T. brucei*. Additionally, limonene did not show significant toxicity in Balb/3T3 mouse fibroblasts (IC_50_ > 100 µg/mL) and exhibited a favourable selectivity index (SI > 17.85). Its trypanocidal activity could be explained by the high degree of unsaturation (two double bonds) in its chemical structure. In addition, its exocyclic methylene group can easily react with SH groups in proteins (e.g., trypanothione synthase), thus enhancing its bioactivity by exposing the parasite to oxidative damage [42]. However, further investigations are required in order to clarify the mechanism of action of essential oil and limonene itself and to evaluate their therapeutic values for trypanosomiasis infections.

## 4. Materials and Methods

### 4.1. Plant Materials

Aerial parts of *E. floribundus* were harvested in Dschang, West Province of Cameroon (1450 m a.s.l.) in February 2013. The plant was identified at the Cameroon National Herbarium (Yaoundé), where a voucher specimen was deposited (5619SRF/Cam). Plant material was dried at room temperature in the shade for one week before undergoing total extraction.

### 4.2. Hydrodistillation

The dry aerial parts (500 g) were reduced into small pieces, then subjected to hydrodistillation in a Clevenger-type apparatus for 4 h using 6 L of deionized water. The essential oil yield (0.2%, *n* = 3) was estimated on a dry-weight basis (*w/w*). Once obtained, the oil was dried (Na_2_SO_4_), transferred into an amber glass flask, and kept at −20 °C before chemical analysis and biological experiments.

### 4.3. Chemicals

For identification of volatiles the following analytical standards were purchased from Sigma-Aldrich (Milan, Italy): α-pinene, β-pinene, *p*-cymene, limonene, γ-terpinene, linalool, *trans*-pinocarveol, terpinen-4-ol, α-terpineol, myrtenol, nerol, carvone, geraniol, geranial, eugenol, (*E*)-caryophyllene, α-humulene, (*E*)-β-farnesene, (*E*)-β-ionone, (*E*)-nerolidol, caryophyllene oxide, *n*-pentacosane, *n*-heptacosane; (*E*)-Phytol was previously isolated from *Onosma echioides* [43]. For retention-index (RI) determination, a mixture of hydrocarbons, ranging from octane (C_8_) to triacontane (C_30_) (Supelco, Bellefonte, PA, USA) was used and run under the experimental conditions reported below. All compounds were of analytical standard grade. Analytical-grade hexane solvent was purchased from Carlo Erba (Milan, Italy); it was successively distilled through a Vigreux column before use.

### 4.4. Chemical Analysis of Essential Oil

GC-MS Analysis was performed on an Agilent 6890N gas chromatograph (Santa Clara, CA, USA) coupled to a 5973N mass spectrometer using two different capillary columns: a HP-5 MS column (5% phenylmethylpolysiloxane, 30 m, 0.25 mm i.d., 0.1-mm film thickness; J & W Scientific (Folsom, CA, USA), and a DB-WAX column (polyethylene glycol; 30 m, 0.25 mm i.d., 0.25-mm film thickness; J & W Scientific,). The oven temperature program was the following: 5 min at 60 °C, subsequently 4 °C/min up to 220 °C, then 11 °C/min up to 280 °C, held for 15 min, for a total run of 65 min. Injector and detector temperatures were 280 °C. He was used as the carrier gas, at a flow rate of 1 mL/min. Split ratio, 1:50; acquisition mass range, *m/z* 29–400. All mass spectra were acquired in electron–impact (EI) mode with an ionization voltage of 70 eV. Oil samples were diluted to 1:100 in *n*-hexane, and the volume injected was 2 µL. Whenever possible, the essential oil constituents were identified by co-injection with authentic standards. Otherwise, the peak assignment was carried out according to the recommendations of the International Organization of the Flavour Industry (http://www.iofi.org/), i.e., by the interactive combination of chromatographic linear retention indices that were consistent with those reported in the literature [22,23,24] for apolar and polar stationary phases, and MS data consisting in computer matching with the WILEY275, NIST 08, ADAMS, FFNSC2 and home-made (based on the analysis of reference oils and commercially available standards) libraries. Quantification of essential oil components was achieved by peak-area internal normalisation without using correction factors.

### 4.5. Antimicrobial Activity

The essential oil of *E. floribundus* was tested against a panel of reference bacterial and yeast species including *Staphylococcus aureus* ATCC 25923, *Escherichia coli* ATCC 25922, *Pseudomonas aeruginosa* ATCC 27853, *Enterococcus faecalis* ATCC 29212, *Candida albicans* ATCC 24433. Extension of the investigation on *S. aureus* was accomplished using four additional *S. aureus* clinical isolates (Table 3) that were resistant to methicillin and maintained in the collection of the Microbiology Unit of the School of Pharmacy at the University of Camerino [25]. Bacterial strains were cultured overnight at 37 °C in blood agar plates. *C. albicans* was grown in Sabouraud Dextrose Agar. Tests by the paper disc diffusion method were performed following the Clinical and Laboratory Standards Institute (CLSI) guidelines [44]. Briefly, a suspension of the tested microorganism (1–2 × 10^8^ cells per mL in saline-10^6^ per mL for Candida) was spread on the solid media plates using a sterile cotton swab. Sterile paper discs (6 mm in diameter) were placed on the surface of inoculated plates and spotted with 10 µL of either the essential oil or 1 mg of extract dissolved in DMSO. The plates were incubated 24 h at 35 ± 1 °C (48 h for *C. albicans*). The diameters of zone inhibition (including the 6 mm disc) were measured with a gauge. A reading of more than 6 mm indicated growth inhibition. No zone inhibition was observed using DMSO alone. Ciprofloxacin (5 μg disc) and Nystatin (100 Units disc) were used as reference antimicrobials against bacteria and fungi, respectively. Each test was repeated at least twice. Antibiotic susceptibility testing by the microdilution method was performed following the international guidelines [44]. Briefly, two-fold serial dilutions of each mixture in Cation Adjusted Mueller Hinton Broth for bacteria and Roswell Park Memorial Institute (RPMI) 1640 for *Candida* were set in 96-well plates starting from 2048 mg/L. An equal volume of the microbial inoculum (10^6^ cfu/mL), obtained by direct colony suspension of an overnight culture, was added to each well of the microtiter plate containing 0.1 mL of the serially diluted test oil/extract. After incubation for 18–24 h at 35 °C, in normal atmosphere, Minimum Inhibitory Concentrations (MICs) were defined as the lowest concentration of compound able to inhibit the growth of the microorganisms. All tests were done in triplicate.

### 4.6. Antioxidant Activity

The antioxidant activity of *E. floribundus* essential oil was measured by using different radical generating systems (DPPH, ABTS and FRAP assay) to assess the free radical scavenging and reducing properties of the oil [45]. All the experiments were conducted in a 96-well microplate assay according to the method of Srinivasan et al. [46], for DPPH assay and following previously-described protocols by Re et al. [47], for measuring the total radical scavenging capacity on ABTS radical. The ferric reducing antioxidant power (FRAP assay) was carried out according to the procedure described by Müller et al. [48], by monitoring the reduction of Fe^3+^-tripyridyl triazine (TPTZ) to blue-coloured Fe^2+^-TPTZ. The ability of *E. floribundus* essential oil to scavenge the different radicals in all three assays was compared to Trolox used as standard. Results as tocopherol-equivalent antioxidant capacity (TEAC) were expressed in μM Trolox equivalents (TE)/g of essential oil.

### 4.7. Cytotoxic Activity on Tumor Cells

A375 (human malignant melanoma cells) and MDA–MB 231 cells (human breast adenocarcinoma cells) were cultured in Dulbecco′s Modified Eagle′s Medium (DMEM) with 2 mM l-glutamine, 100 IU/mL penicillin, 100 µg/mL streptomycin, and supplemented with 10% heat-inactivated fetal bovine serum (HI-FBS). HCT116 cells (human colon carcinoma cells), were cultured in RPMI1640 medium with 2 mM l-glutamine, 100 IU/mL penicillin, 100 μg/mL streptomycin, and supplemented with 10% HI-FBS. Cells were cultured in a humidified atmosphere at 37 °C in presence of 5% CO_2_. The MTT assay was used as a relative measure of cell viability. Cell-viability assays were carried out as described [49]. Briefly, cells were seeded at the density of 2 × 10^4^ cells/mL. After 24 h, samples were exposed to different concentrations of essential oil (0.78–200 μg/mL). Cells were incubated for 72 h in a humidified atmosphere of 5% CO_2_ at 37 °C. Cisplatin was used as the positive control (0.05–20 µg/mL). At the end of incubation, each well received 10 µL of 3-(4,5-dimethyl-2-thiazolyl)-2,5-diphenyl-2H-tetrazoliumbromide (MTT) (5 mg/mL in phosphate-buffered saline, PBS) and the plates were incubated for 4 h at 37 °C. The extent of MTT reduction was measured spectrophotometrically at 540 nm using a Titertek Multiscan microElisa (Labsystems, Helsinki, Finland. Experiments were conducted in triplicate. Cytotoxicity is expressed as the concentration of compound inhibiting cell growth by 50% (IC_50_). The IC_50_ values were determined with the GraphPad Prism 4 computer program (GraphPad Software, San Diego, CA, USA).

### 4.8. Enzyme Inhibition Assay

Recombinant *S. aureus* NadD, available in our laboratory, was used as a representative bacterial target of the NadD enzyme family. Previous reports, indeed, have proven that this target enzyme is highly conserved in bacteria [17] and small molecule inhibitors may be simultaneously active on NadDs from distant species such as *S. aureus* and *M. tuberculosis* [50]. Murine NMNAT1 was chosen as the functionally equivalent mammalian enzyme [51].

The enzyme inhibition assay setup for the bacterial enzyme and the mammalian counter target is described in detail in Vitali et al. [27]. Briefly, enzyme rates and inhibition were measured by an established endpoint assay based on the detection at 620 nm of the phosphate byproduct formed [17]. A continuous assay based on detection at 340 nm of the reduced form of nicotinamide adenin dinucleotide (NADH) formed was used as an alternate assay [52].

### 4.9. T. brucei and Mammalian Cell Culture

*T. brucei* BSFs (TC221) were cultured at 37 °C with 5% CO_2_ in HMI-9 medium supplemented with 10% (*v/v*) heat-inactivated fetal bovine serum (Thermo Fisher Scientific [Gibco], Waltham, MA, USA). Mouse BALB/3T3 fibroblast (ATCC no CCL-163) were cultivated at 37 °C and 5% CO_2_ in Dulbecco′s modified Eagle′s medium (Sigma-Aldrich), supplemented with 10% (*v/v*) heat-inactivated fetal bovine serum, glutamine (0.584 g/L) and 10 mL/L 100× penicillin-streptomycin (Thermo Fisher Scientific [Gibco]) [53].

### 4.10. Growth Inhibition Assay on T. brucei and Balb/3T3 Cells

The essential oil was dissolved in dimethyl sulfoxide (DMSO). It was serially diluted with growth medium to concentrations ranging from 2 × 10^−5^ to 200 µg/mL in 96-well microtiter plates (100 µL/well). Subsequently, 100 µL of *T. brucei* or mammalian cell culture was added to each well (20,000 cells/well). After 48 h incubation, the plates were treated for 24 h with 20 µL of resazurine (Sigma-Aldrich). They were subsequently quantified by fluorescence (540 nm excitation and 590 nm emission) using an Infinite M200 microplate reader (Tecan Group Ltd., Männedorf, Switzerland). The IC_50_ values were calculated by fitting the data to a log inhibitor vs. response curve (variable slope, four parameters) using the GraphPad Prism version 5.04 software. 

## 5. Conclusions

The present work provides new insights into the phytochemical and biological properties of *E. floribundus*. The essential oil of this plant revealed a good potential as bacterial NadD inhibitor, thus being a promising candidate for antibiotic formulations. The exhibited trypanocidal activity, on one hand, confirmed partially the traditional use in the treatment of protozoal diseases, and on the other hand, makes it a potential candidate for standardized herbal medicines against trypanosomiasis that are highly requested in developing countries with poor medical infrastructure and difficult access to conventional medical treatments.

## Figures and Tables

**Table 1:** Composition of the essential oil hydrodistilled from the aerial parts of *Erigeron floribundus*.

N.	Component ^1^	RI Calc. ^2^		RI Lit. ^3^		% ^4^	ID ^5^
		HP-5MS	DBWAX	Apolar	Polar		
1	butyl methyl ketone	780	1088	786	1088	tr ^6^	RI, MS
2	hexanal	792	1024	801	1024	0.1	RI, MS
3	(3*E*)-hexenol	844	1371	844	1371	0.2	RI, MS
4	*n*-hexanol	858	1363	863	1363	0.1	RI, MS
5	1-(2-methyl-2-cyclopenten-1-yl)-ethanone	871				tr	MS
6	α-pinene	921	1026	932	1026	0.2	Std
7	sabinene	960	1125	969	1125	tr	RI, MS
8	β-pinene	962	1112	974	1112	2.1	Std
9	myrcene	983	1167	988	1165	0.1	Std
10	2-pentyl furan	983	1231	984	1231	0.1	RI, MS
11	*p*-cymene	1017	1271	1020	1272	tr	Std
12	limonene	1020	1199	1024	1199	8.8	Std
13	(*E*)-β-ocimene	1042	1255	1044	1255	0.1	RI, MS
14	γ-terpinene	1051	1253	1054	1255	tr	Std
15	terpinolene	1080	1283	1086	1282	tr	RI, MS
16	linalool	1096	1555	1095	1556	0.1	Std
17	*n*-nonanal	1102	1393	1100	1395	tr	RI, MS
18	(*E*)-4,8-dimethyl-1,3,7-nonatriene	1112				tr	MS
19	*trans*-*p*-mentha-2,8-dien-1-ol	1114		1119		tr	RI, MS
20	*trans*-pinocarveol	1129	1656	1135	1657	0.1	Std
21	nerol oxide	1150		1154		tr	RI, MS
22	pinocarvone	1153	1563	1160	1565	0.1	RI, MS
23	lavandulol	1163	1686	1170	1686	0.1	RI, MS
24	terpinen-4-ol	1169	1600	1174	1600	0.1	Std
25	*trans*-isocarveol	1180	1838	1175		tr	RI, MS
26	α-terpineol	1182	1688	1186	1690	0.2	Std
27	myrtenal	1188	1648	1195	1648	0.1	RI, MS
28	myrtenol	1188	1794	1194	1794	0.1	Std
29	β-cyclocitral	1212		1217		0.1	RI, MS
30	nerol	1223	1840	1227	1838	0.4	Std
31	carvone	1236	1744	1239	1745	0.1	Std
32	geraniol	1253	1850	1249	1852	tr	Std
33	perilla aldehyde	1265	1783	1265	1784	tr	RI, MS
34	geranial	1268	1730	1264	1730	tr	Std
35	perilla alcohol	1291	2028	1290	2029	0.1	RI, MS
36	silphiperfol-5-ene	1310	1421	1326		0.3	RI, MS
37	α-cubebene	1338	1457	1345		0.1	RI, MS
38	eugenol	1351	2164	1356	2167	0.1	Std
39	α-copaene	1362	1488	1374	1488	0.7	RI, MS
40	modheph-2-ene	1363	1516	1377		0.7	RI, MS
41	α-isocomene	1370	1529	1377		0.5	RI, MS
42	β-cubebene	1378		1379		0.2	RI, MS
43	β-elemene	1380		1389		0.2	RI, MS
44	β-isocomene	1387		1407		0.4	RI, MS
45	(*Z*)-caryophyllene	1392	1570	1408		2.3	RI, MS
46	(*E*)-caryophyllene	1403	1590	1417	1585	4.2	Std
47	β-copaene	1414	1535	1430		0.4	RI, MS
48	β-gurjunene	1422		1431		0.1	RI, MS
49	α-*trans*-bergamotene	1425	1583	1432	1586	2.1	RI, MS
50	α-humulene	1437	1663	1452	1665	1.0	Std
51	geranyl acetone	1447		1453		0.1	RI, MS
52	(*E*)-β-farnesene	1451	1745	1454	1745	5.5	Std
53	γ-muurolene	1464	1683	1478		0.4	RI, MS
54	germacrene D	1466	1701	1484	1700	0.1	RI, MS
55	β-selinene	1470		1489		0.1	RI, MS
56	*ar*-curcumene	1473	1770	1479	1769	1.0	RI, MS
57	(*E*)-β-ionone	1476	1934	1487	1936	0.1	Std
58	*cis*-β-guaiene	1481		1492		0.2	RI, MS
59	γ-curcumene	1481	1673	1481		0.2	RI, MS
60	α-muurolene	1487	1719	1500		0.2	RI, MS
61	modhephen-8-β-ol	1492		1513		0.2	RI, MS
62	γ-cadinene	1499		1513		0.3	RI, MS
63	δ-cadinene	1511	1753	1522	1752	0.5	RI, MS
64	α-calacorene	1528	1907	1544		0.1	RI, MS
65	(*E*)-nerolidol	1557	2048	1561	2049	2.1	Std
66	spathulenol	1564	2127	1577	2132	12.2	RI, MS
67	caryophyllene oxide	1567	1977	1582	1983	12.4	Std
68	salvial-4(14)-en-1-one	1577	2000	1594		0.8	RI, MS
69	humulene epoxide II	1591	2034	1608	2040	1.7	RI, MS
70	caryophylla-4(12),8(13)-dien-5α-ol	1615		1639		0.6	RI, MS
71	caryophylla-4(12),8(13)-dien-5β-ol	1619		1639		1.0	RI, MS
72	*tau*-cadinol	1626	2126	1625	2125	1.0	RI, MS
73	muurola-4,10(14)-dien-1-beta-ol	1637		1630		1.0	RI, MS
74	α-cadinol	1640	2232	1652	2232	1.0	RI, MS
75	14-hydroxy-9-*epi*-(*E*)-caryophyllene	1656		1668		2.1	RI, MS
76	germacra-4(15),5,10(14)-trien-1-α-ol	1669		1685		2.1	RI, MS
77	pentadecanal	1706	2041	1705	2041	0.6	RI, MS
78	β-bisabolenol	1776		1785		0.2	RI, MS
79	14-hydroxy-δ-cadinene	1787		1788		0.3	RI, MS
80	neophytadiene	1831				0.2	MS
81	2-pentadecanone, 6,10,14-trimethyl-	1837	2131	1838	2131	0.3	RI, MS
82	hexadecanoic acid	1962	2918	1959	2917	2.5	RI, MS
83	(*E*)-phytol	2094	2624	2096	2622	0.7	Std
84	*n*-pentacosane	2500	2500	2500	2500	tr	Std
85	*n*-heptacosane	2700	2700	2700	2700	tr	Std
	Total identified (%)					78.6	
	Grouped compounds						
	Monoterpene hydrocarbons					11.5	
	Oxygenated monoterpenes					1.5	
	Sesquiterpene hydrocarbons					21.9	
	Oxygenated sesquiterpenes					38.5	
	Others					5.2	

^1^ Compounds are listed in order of their elution from an HP-5MS column. Their nomenclature was in accordance with Adams [22]. ^2^ Linear retention index on HP-5MS and DB-Wax column, experimentally determined using homologous series of C8–C30 alkanes. ^3^ Linear retention index taken from Adams [22] and/or NIST08 [23] for apolar columns, and from FFNSC2 [24] and/or NIST08 for polar columns. ^4^ Percentage values are means of three determinations with an RSD% for the main components below 5% in all cases. ^5^ Identification methods: std, based on comparison with authentic compounds; MS based on comparison with Wiley. ADAMS and NIST08 MS database; RI, based on comparison of RI with those reported in ADAMS and NIST08. ^6^ tr, traces (mean value below 0.1%).

**Table 2:** In vitro growth inhibition of tumor cells by *Erigeron floribundus* essential oil.

	Cell Line (IC_50_ µg/mL) ^1^		
	A375 ^2^	MDA–MB 231 ^3^	HCT116 ^4^
Essential oil	22.5	20.8	14.9
95% C.I. ^5^	20.6–24.6	16.82–25.77	13.10–17.00
Positive control			
Cisplatin	0.5	2.5	2.5
95% C.I.	0.4–0.5	2.0–3.0	2.2–2.9

^1^ IC_50_ = the concentration of compound that affords a 50% reduction in cell growth (after 72 h of incubation). ^2^ Human malignant melanoma cell line. ^3^ Human breast adenocarcinoma cell line. ^4^ Human colon carcinoma cell line. ^5^ C.I. Confidence interval.

**Table 3:** In vitro antimicrobial activity of *Erigeron floribundus* essential oil.

Species	Strain	Essential Oil		Reference Antibiotic ^1^	
		IZD ^2^ (mm)	MIC ^3^ (µg/mL)	IZD (mm)	MIC (µg/mL)
*S. aureus*	ATCC 29213	14.3 ± 0.6	512	20.67 ± 1.15	0.25
	MRSA ^4^ -19		1024–2048		
	MRSA-42		512–1024		
	MRSA-75		512–1024		
	MRSA-101		512–1024		
*E. faecalis*	ATCC 29212	8.7 ± 0.6	4096	24.0 ± 2.6	0.5–1
*E. coli*	ATCC 25922	n.a. ^5^	N.D. ^6^	19.7 ± 0.6	0.016
*P. aeruginosa*	ATCC 27853	n.a.	N.D.	21.7 ± 0.6	0.5
*C. albicans*	ATCC 24433	9.0 ± 1.0	512	16.33 ± 0.58	4.0

^1^ Ciprofloxacin for Gram-positive species, gentamicin for Gram-negative ones; nystatin for *C. albicans*. ^2^ IZD, inhibition zone diameter. Each value is an average of three independent experiments with standard errors indicated. ^3^ MIC; minimum inhibitory concentration. ^4^ MRSA, Methicillin-Resistant *Staphylococcus aureus.* ^5^ n.a., no activity. ^6^ N.D., not determined.

**Table 4:** Antioxidant activity of *Erigeron floribundus* essential oil.

	DPPH		ABTS		FRAP
	TEAC ^1^ μmol·TE/g	IC_50_^2^ μg/mL	TEAC ^1^ μmol·TE/g	IC_50_^2^ μg/mL	TEAC ^1^ μmol·TE/g
*E. floribundus* oil	16.9 ± 0.1	356.7 ± 7.1	85.5 ± 3.6	74.9 ± 3.2	411.9 ± 2.40
Trolox		1.51 ± 0.04		1.6 ± 0.01	

^1^ TEAC: Trolox equivalent (TE) antioxidant concentration. ^2^ IC_50_: The concentration giving a reduction of 50%. Each value is an average of three independent experiments with standard errors indicated.

**Table 5:** Inhibition of *T. brucei* and Balb/3T3 fibroblast proliferation.

	*T. brucei* IC_50_^1^ (μg/mL)	Balb/3T3 IC_50_^1^ (μg/mL)	Selectivity Index (SI)
*E. floribundus* oil	33.5 ± 2.7	>200	>5.97
Carophyllene oxide	>200	N.D.	
Limonene	5.6 ± 1.6	>100	>17.85

^1^ Each value is an average of three independent experiments with standard errors indicated.
